# Supplementary material for: Quantification of permethrin resistance and kdr alleles in Florida strains of Aedes aegypti (L.) and Aedes albopictus (Skuse)
Source: PLoS Negl Trop Dis. 2018 Oct 24;12(10):e0006544. doi: 10.1371/journal.pntd.0006544 (PMC6218098; doi:10.1371/journal.pntd.0006544)
Supplement: S2 File — (DOCX) [file pntd.0006544.s002.docx]

Supplemental File 2: Comparison of 1016 and 1534 SNP frequencies between egg and adult stages of *Aedes aegypti* strains by *kdr* melt curve analysis

Location/generation 1016 lifestage 1534 n

VV VI II FF FC CC

Jacksonville-5 F1 0.10 0.51 0.39 egg 0.00 0.00 1.00 86

Jacksonville-5 F1 0.13 0.45 0.42 adult 0.00 0.04 0.96 85

Riverside, Jax F1 0.20 0.48 0.32 egg 0.00 0.00 1.00 77

Riverside, Jax F1 0.33 0.22 0.44 adult 0.00 0.00 1.00 63

Miami Beach F1 0.01 0.11 0.88 egg 0.00 0.01 0.98 90

Miami Beach F1 0.00 0.06 0.94 adult 0.00 0.00 1.00 90

Alt. Spgs., FL F1 0.20 0.45 0.34 egg 0.03 0.01 0.95 86

Alt. Spgs., FL F1 0.19 0.54 0.27 adult 0.03 0.01 0.95 86

Winter Park, FL F1 0.00 0.24 0.76 egg 0.00 0.00 1.00 89

Winter Park, FL F1 0.05 0.19 0.75 adult 0.02 0.02 0.96 73
